# A psychometric evaluation of the interRAI Child and Youth Mental Health instruments (ChYMH) anxiety scale in children with and without developmental disabilities

**DOI:** 10.1186/s12888-020-02785-9

**Published:** 2020-07-29

**Authors:** S. L. Stewart, S. E. Babcock, Y. Li, H. P. Dave

**Affiliations:** 1 The University of Western Ontario, Faculty of Education, John George Althouse Building | 1137 Western Road, London, Ontario N6G 1G7 Canada; 2 Department of Psychology, The University of Western Ontario, Social Sciences Building | 1151 Richmond St, London, Ontario N6A 3K7 Canada

**Keywords:** Anxiety, Mental health, Intellectual and developmental disabilities, Assessment, Child & youth, InterRAI

## Abstract

**Background:**

With 10 to 20% of Canadian children suffering with mental illness, the importance of early identification and accurate assessment systems is clear. Unfortunately, many do not receive the mental health treatment necessary and wait-times for assessment can span up to a year. In response, the interRAI suite of assessments were designed to comprehensively assess early signs of mental health impairments in children from birth to 18 years.

**Methods:**

This study assesses the psychometric properties of the Anxiety Scale and addresses the identification of anxiety within children diagnosed with intellectual and developmental disabilities (IDD); a commonly underrepresented sample in mental health psychometric studies. Data was collected from children aged 4–18 years in three different samples.

**Results:**

Results indicated reliable internal consistency and factor structure, as well as moderate-to-strong convergent validity.

**Conclusions:**

We conclude that the Anxiety Scale exhibits psychometric qualities which demonstrate its clinical utility for use within a child sample, as well as in children with IDD. The findings provide support to a larger body of research which show consistent psychometric rigour of the interRAI measures.

## Background

With 10 to 20% of Canadian children and youth (hereafter both referred to as children) currently suffering from mental illness [1], the importance of early clinical identification is of paramount importance [2]. Specifically, results from Canada’s National Longitudinal Survey of Children and Youth (NLSCY) indicate the prevalence of anxiety problems as ranging from 2 to 12%. Most adult psychiatric disorders originate in childhood and show stability, persistence, and long-term aversive outcomes in adulthood [2–4]. Specifically, there is large-scale longitudinal evidence which suggests that early childhood diagnosis (as early as 3 years old) can predict later mental health diagnoses by five-fold, in addition to homotypic and heterotypic continuity [5].

Acknowledging the chronicity and devastating economic impact of long-term mental health issues [6], it is important to have an accurate assessment system of childhood psychiatric symptoms. Unfortunately, less than 75% of children receive the mental health treatment they need and wait-times for assessment at service centres range from 6 months to 1 year [7–9]. In addition, assessment of co-morbid psychiatric disorders is an exceptionally complicated problem in children with intellectual disability, who represent a non-trivial (1–3%) portion of the population [10]. A study by the Office of National Statistics in Great Britain found that children with intellectual disabilities are six-times more likely than the general population to have one or more co-morbid psychiatric disorders (e.g., anxiety, attention-deficit hyperactivity disorder, depressive disorders, conduct disorders) [11], as well as experience somatic complaints, aggressive behaviour, anxiety, and other internalizing and externalizing problems [12]. Approximately 30–50% of children with intellectual disability have a mental health disorder, compared to 8–18% for those without [13]. Further complicating the challenges for children and their families is a dearth of appropriate assessment tools for this unique population. Many assessments have a narrow focus, which may omit areas of need that could be captured through comprehensive assessment [14, 15]. Further, there is inconsistent use of assessment instruments across organizations, with many using tools that have not undergone systematic psychometric evaluation [16]. Consequently, clients may be triaged based on multiple instruments that lack well-established reliability and validity [17].

The primary difficulty when assessing children with co-morbid mental health issues and/or developmental disabilities is the variability inherent in their physical, emotional, and intellectual development [18], particularly with respect to anxiety. Anxiety-related disorders are often severe and chronic, frequently stemming from early childhood and persisting into later stages of life [19–21]. Diagnosis is frequently based on an abnormal pattern of presentation of symptoms (e.g., headache, sweating, and excessive worry) and there is a lack of consensus on its etiology [22]. However, it is the most common mental health disorder among children [23] and therefore warrants research into efficiency of its assessment. A child’s anxiety can vary with the environmental context; as a result, behaviours can be misunderstood or difficult to interpret [24]. Specifically, as children experience more complex social milieus, they often become aware of their differences, resulting in increasingly high levels of anxiety and emotional stress [25]. These issues contribute to frustration when attempting to access appropriate services, particularly for children with complex service needs [26, 27].

The need for effective assessment systems which provide a comprehensive and scientifically rigorous approach to mental health assessment led to the development of an assessment system incorporating a lifespan approach by interRAI, a non-profit, international organization network of 100 members from over 35 countries [28]. A unique feature of the interRAI assessment instruments is their integrated use along the service continuum (e.g., homecare, palliative care, emergency department) and intellectual level; they are designed to monitor symptoms and treatment outcomes across a wide range of age groups and vulnerable populations [29]. The instruments have strong psychometric properties and criterion validity in adult and geriatric samples [30, 31], children/youth samples [32–36], and across different cultures [37, 38].

One of the newest assessment suites available in the interRAI family of assessments are the *child and youth instruments,* designed to comprehensively assess early signs of mental health impairments in children from birth to 18 years [39–42]. Assessments are conducted through a variety of sources, including: communication with the primary caregiver, observation of the child, communication with healthcare providers, and review of medical records. The interRAI Child and Youth Mental Health Instrument (ChYMH) and the interRAI Child and Youth Mental Health and Developmental Disability (ChYMH-DD) instruments are comprised of over 400 items that assess a range of variables relevant to the physical and mental health of children [39, 40]. They assess pediatric mental health in both a dimensional and holistic framework, encouraging clinicians to understand both the individual and environmental context of the child. Specifically, information collected includes the child’s relationship with family (e.g., parenting), home environment, stress and trauma (e.g., abuse), childcare services, medications, physical health problems, treatment service utilization (e.g., self-care skills training), neuropsychological development (e.g., motor skills), communication abilities (e.g., ability to understand, ability to be understood), as well as socioemotional and behavioural skills (e.g., control of anger). In addition, the instruments also have collaborative action plans (CAPs) embedded in the instrument to provide real-time, evidence-informed recommendations for care-planning, particularly around areas of risk [39, 40, 43, 44].

### Rationale for current study

The suite of interRAI Child and Youth assessments are widely used in Ontario, Canada and are now being implemented across multiple countries (e.g., United States, Netherlands, Finland). Given the importance of early identification of anxiety-related problems in children at varying levels of functioning, the present study aims to explore the factorial structure, reliability, and validity of the Anxiety Scale embedded within the interRAI Child and Youth suite among three samples: children referred for mental health services from high risk schools, children receiving mental health services and children receiving mental health services who have developmental/intellectual disabilities. This scale is embedded within a variety of instruments within the interRAI child and youth suite of instruments; therefore, it is important that the individual components are performing as expected to facilitate appropriate screening and triaging of children with mental health concerns. Moreover, the present study addresses the identification of anxiety within a subgroup of children with diagnosed developmental/intellectual disability, addressing a key piece of the literature where there is lacking available information on the prevalence of co-morbid developmental/intellectual and mental health concerns.

## Methods

### Data collection

We examined data from three independent samples of children, each of whom completed one of the three following instruments: the interRAI Child and Youth Mental Health assessment (ChYMH) [39], the interRAI Child and Youth Mental Health and Developmental Disability assessment (ChYMH-DD) [40], or the interRAI Child and Youth Mental Health-Screener assessment (ChYMH-Screener) [41], which is a revised, shorter screening instrument developed based on the ChYMH. Assessors who had at least 2 years of experience working with children with mental health issues received a 2.5-day intensive training on administering the assessments and completed all assessments. In a validation effort, two well-established external criterion measures were also administered to a subset of the children included in this study. The Child Behaviour Checklist (CBCL) [45] was completed for a subsample of children in the ChYMH sample, and the Behaviour Assessment System for Children, Third Edition (BASC-3) [46] was completed with children in the ChYMH-Screener sample. Only a subset of children in the study were administered these validation measures, and therefore, only this subset data was available for analysis.

#### Data collection for the ChYMH sample

Over 6000 clinically referred children between the ages of 4 and 18 years were assessed using the ChYMH assessment tool. Data from these children were collected from 46 mental health agencies across Ontario, Canada. Primary caregivers for 57 children independently completed the CBCL assessments within 3 days of the initial ChYMH assessments. These 57 families were selected because data was available on both the CBCL and ChYMH assessment instruments within the allocated 3-day timeframe as part of a previous validation study.

#### Data collection for the ChYMH-DD sample

A total of 657 children between the ages of 4 to 18 years were assessed using the ChYMH-DD assessment tool at participating mental health agencies. The ChYMH-DD assessments were completed by trained assessors at four agencies that provided mental health services to children with developmental delays or intellectual disabilities in the province of Ontario, Canada.

#### Data collection for the ChYMH-screener sample

A total of 79 children aged 4 to 14 years were assessed using the ChYMH-Screener. These children were recruited from various high-risk schools in a southwest city of Ontario, Canada. The ChYMH-Screener assessments were administered by trained assessors. The BASC-3 [46] was also administered to this sample, and was independently completed by the child’s primary caregiver within 3 days of the initial ChYMH-Screener assessment. Both BASC-3 and ChYMH data was only available for 79 participants within the allocated 3-day timeframe as part of a previous validation study.

#### Ethics approval

Data collections for the ChYMH (REB # 106415), ChYMH-DD (REB 106415), and ChYMH-Screener (REB # 106741) samples were all approved by the University of Western Ontario research ethics board.

### Measures

#### interRAI anxiety subscale

The interRAI Anxiety Scale consists of six items and aims to capture the frequency of several anxiety symptoms of the child. Such symptoms include: *have anxious complaints or concerns, unrealistic fears, obsessive thoughts, intrusive thoughts or flashbacks, episodes of panic,* and *nightmares*. The items are scored as 0 (“Not present”), 1 (“Present but not exhibited in last 3 days”), 2 (“Exhibited on 1-2 of last 3 days”), and 3 (“Exhibited daily in last 3 days, 1-2 episodes”), or 4 (“Exhibited daily in last 3 days, 3 or more episodes or continuously”). Higher scores on the Anxiety Scale therefore indicate higher levels of anxiety. This scale is embedded in all of the assessment instruments utilized in this study (the ChYMH, the ChYMH-DD, and the ChYMH-Screener).

#### CBCL-internalizing

The CBCL provides evaluations of both internalizing and externalizing symptoms for a child. It is comprised of 120 items which are rated on a three-point scale (0 = *not true*; 1 = *somewhat or sometimes true*; 2 = *very often true*). The internalizing scale captures syndromes such as Withdrawn/depressed, Anxious/depressed, and Somatic Complaints, whereas the externalizing scale of the CBCL captures syndromes such as Rule-breaking and Aggressive behaviours. The CBCL is widely used as a measure of children’s behavioural and emotional problems and has strong empirical support for its reliability and validity [47]. In the current study, the *internalizing* scale of the CBCL was used as an established external criterion, against which the convergent validity of the Anxiety Scale was tested.

#### DSM-IV provisional diagnosis-anxiety disorder

As children in the ChYMH-DD sample were not administered any validity measures, their diagnostic data on anxiety disorders were used as the convergent criterion measure. The ChYMH-DD includes a subsection, *diagnostic and other health information,* which collects a child’s information on 12 provisional including an anxiety disorder as per the Diagnostic and Statistical Manual of Mental Disorders, Fourth Edition, Text Revision (DSM-IV-TR) [48]. These diagnoses were determined by a psychiatrist, psychologist, or attending physician. All applicable diagnoses were ranked for the importance as a contributing factor to a child’s reason for service. In the current study, the ranking data were recoded into either “Presence” or “Absence” of a DSM-IV Anxiety Disorder diagnosis.

#### BASC-3 anxiety

Another widely used assessment for the behavioural and emotional disorders experienced among children is the Behaviour Assessment System for Children (BASC). The BASC allows the child’s information to be collected from multiple sources using different components such as the Teacher Rating Scale, Parent Rating Scale, Self-Report of Personality, Structured Development History, and Student Observation System. The BASC has good reliability indices for its different components [49, 50], and good validity as a measure of children’s behaviours [51, 52]. The Parent Rating Scale from the third edition of the BASC was used in this current study (BASC-3), and children’s standardized scores on the BASC-3 *Anxiety Scale* were compared to their Anxiety Scale scores on the interRAI child and youth instruments. The BASC-3 Anxiety Scale is comprised of 13 items assessing nervousness, generalized fears, and worries that are typically irrational. Three of the items are rated on a dichotomous scale (0 – True; 2 – False). An example item includes: “*I can never seem to relax*”. The other ten items are rated on a 4-point Likert Scale ranging from 0 (*Never*) to 3 (*Almost Always*). Example items here include: “*I get so nervous I can’t breathe*” and “*I worry when I go to bed at night.*” The score ranges from 0 to 36.

### Statistical analyses

Only children with valid responses on all the Anxiety Scale items in the samples were included in the analyses for the current study. The ChYMH and ChYMH-DD samples were utilized to test the scale reliability. To test the convergent validity, the Anxiety Scale was tested against the CBCL internalizing scores, the DSM-IV anxiety disorder diagnosis, and the BASC-3 anxiety t-scores, utilizing the ChYMH subsample, the ChYMH-DD sample, and the ChYMH-Screener sample, respectively.

#### Scale reliability

To assess the inter-item reliability of the Anxiety Scale, polychoric correlations were calculated for the scale items, together with Cronbach’s alpha based on polychoric correlations. Polychoric correlations provide estimations of the association between two variables that are continuously distributed but measured categorically or ordinally [53]. Polychoric correlations have been found to provide more accurate estimates of pair-wise correlations and factor loadings when the continuous variables are measured as categorical data, when compared to Pearson’s product-moment, Spearman’s rho, and Kendall’s tau-*b* correlations [54]. The inter-item correlations and Cronbach’s alpha that are calculated using polychoric correlations can be interpreted in the same way as those based on Pearson’s correlations [54]. In addition, we conducted a robust unrestricted factor analysis [55, 56] using the diagonally weighted least squares (DWSL) method, which has been shown to provide relatively accurate estimates with ordinal data [57]. The factor analysis was also based on the polychoric correlation matrix of the items. Five thousand bootstrap samples were used to estimate the asymptotic covariance matrix. The FACTOR software, Version 10.4.01 [58] was used to carry out all the previously described analyses based on polychoric correlations.

#### Convergent validity

Three sets of analyses were used to establish the convergent validity of the Anxiety Scale. First, the ChYMH subsample was divided into two groups using their CBCL internalizing scores: the ones with a *t*-score above 70 were categorized as falling into the clinical range, whereas those with a *t*-score below 70 were considered as subclinical [45]. The cut-off score of 70 corresponds to any score above the 97th percentile and identified as within the clinical range. Those below a score of 70 fall into either the normal or borderline categories. A score within the clinical range for the higher-order internalizing domain incorporates anxiety as one of the major features. These children’s scores on the Anxiety Scale were then used in a receiver operating characteristic (ROC) curve analysis [59] to predict their CBCL internalizing group membership.

Second, the convergent validity of the Anxiety Scale among children with developmental delays and intellectual disabilities was tested against their DSM-IV anxiety disorder diagnosis. Children’s scores on the Anxiety Scale were used in a ROC analysis to predict whether they had an anxiety disorder diagnosis in the ChYMH-DD sample.

Finally, using the ChYMH-Screener sample, children’s scores on the Anxiety scale were correlated with their standardized scores on the BASC-3 Anxiety scale. All the validation analyses were carried out using SPSS version 23.0.

## Results

### Sample characteristics

#### ChYMH sample

A total of 6086 children (59.8% male), aged between 4 and 18 years (*M =* 11.98, *SD* = 3.60), from the ChYMH sample had responses on all the Anxiety Scale items. See Table 1 for the demographic information of children included in the analyses from the ChYMH sample. The total scores on the Anxiety Scale range from 0 to 24, with a mean of 5.18 (*SD* = 4.84), skewness = 1.02 (*SE* = .03), kurtosis = 0.56 (*SE* = .06). See Table 2 for means and standard deviations of children’s scores on the Anxiety Scale by gender and age groups for all the samples.

**Table 1:** Demographic information for the children included in the analyses from the ChYMH sample

	N (% of all the children included in the analyses)		
Gender			
Male	3640 (59.8%)		
Female	2446 (40.2%)		
Patient Type			
Inpatient	508 (8.3%)		
Outpatient	5578 (91.7%)		
	N (%)	Presence (%)	Absence (%)
DSM-IV Provisional Diagnosis			
Reactive Attachment Disorder	4534 (74.5%)	135 (2.2%)	4399 (72.3%)
Attention Deficit Hyperactivity Disorder	4800 (78.9%)	2524 (41.5%)	2276 (37.4%)
Disruptive Behaviour Disorder	4660 (76.6%)	1276 (21.0%)	3384 (55.6%)
Learning or Communication Disorder	4634 (76.1%)	1204 (19.8%)	3430 (56.4%)
Autism Spectrum Disorder	4537 (74.5%)	559 (9.2%)	3978 (65.4%)
Substance-related disorders	4619 (75.9%)	155 (2.5%)	4464 (73.3%)
Schizophrenia and other psychotic disorders	4639 (76.2%)	36 (0.6%)	46.3 (75.6%)
Mood disorders	4460 (73.3%)	921 (15.1%)	3539 (58.1%)
Anxiety disorders	4534 (74.5%)	2088 (34.3%)	2446 (40.2%)
Eating disorder	4624 (76.0%)	99 (1.6%)	4525 (74.4%)
Sleep disorders	4588 (75.4%)	165 (2.7%)	4423 (72.7%)
Adjustment disorders	4554 (74.8%)	177 (2.9%)	4377 (71.9%)

Information regarding DSM-IV Provisional Diagnosis was not available for children who had not been seen by a Psychiatrist prior to the assessment

**Table 2:** Means and Standard Deviations of Total Scores on the Anxiety Scale by Age Group and Gender

Age Group	Gender	Samples Mean (*Standard Deviation*)		
		ChYMH	ChYMH-DD	ChYMH-screener
Age 7 and Under	Male	4.29 (*4.41*)	3.57 (*3.77*)	4.60 (*3.68*)
	Female	4.97 (*4.70*)	3.04 (*3.12*)	2.47 (*2.97*)
Age 8 to 11	Male	5.49 (*4.88*)	4.81 (*4.09*)	5.29 (*5.05*)
	Female	5.37 (*5.08*)	5.87 (*4.72*)	3.78 (*4.37*)
Age 12 and Above	Male	4.50 (*4.42*)	5.65 (*4.42*)	3.80 (3.63)
	Female	5.93 (*5.15*)	5.70 (*4.66*)	3.63 (4.23)

#### ChYMH-DD sample

The ChYMH-DD sample consisted of 657 children (73.2% male), aged between 4 and 18 years (*M* = 11.88, *SD* = 3.78). See Table 3 for the demographic information of children included in the analyses from the ChYMH-DD sample. Among these children, the total scores on the Anxiety Scale range from 0 to 22, with a mean of 5.16 (*SD* = 4.36), skewness of 0.92 (*SE* = .10), and kurtosis of 0.61 (*SE* = .19).

**Table 3:** Demographic information for the children included in the analyses from the ChYMH-DD sample

	N (% of all the children included in the analyses)		
Gender			
Male	481 (73.2%)		
Female	176 (26.81%)		
Patient Type			
Inpatient	103 (15.7%)		
Outpatient	554 (84.3%)		
	N (%)	Presence (%)	Absence (%)
DSM-IV Provisional Diagnosis			
Reactive Attachment Disorder	560 (85.2%)	12 (2.1%)	548 (83.4%)
Attention Deficit Hyperactivity Disorder	541 (82.3%)	219 (33.3%)	322 (49.0%)
Disruptive Behaviour Disorder	548 (83.4%)	92 (14.0%)	456 (69.4%)
Learning or Communication Disorder	558 (84.9%)	292 (44.4%)	266 (40.5%)
Autism Spectrum Disorder	569 (86.6%)	297 (45.2%)	272 (41.4%)
Substance-related disorders	564 (85.8%)	15 (2.3%)	549 (83.6%)
Schizophrenia and other psychotic disorders	556 (84.6%)	8 (1.2%)	548 (83.4%)
Mood disorders	549 (83.6%)	23 (3.5%)	526 (80.1%)
Anxiety disorders	528 (80.4%)	116 (17.7%)	412 (62.7%)
Eating disorder	561 (85.4%)	7 (1.1%)	554 (84.3%)
Sleep disorders	554 (84.3%)	23 (3.5%)	531 (80.8%)
Adjustment disorders	561 (85.4%)	9 (1.4%)	552 (84.0%)

Information regarding DSM-IV Provisional Diagnosis was not available for children who had not been seen by a Psychiatrist prior to the assessment

#### ChYMH-screener sample

The ChYMH-screener sample consisted of 79 children (51.9% Male) aged from 4 to 14 years (*M* = 8.33, *SD* = 2.58). The screener sample is not represented in the demographic data tables as it does not include any DSM items and therefore, such information for this sample is unavailable.^1^

### Scale reliability

The reliability of the Anxiety Scale was examined using both the ChYMH sample and the ChYMH-DD sample. Both samples generated comparable results that indicate good reliability (as per guidelines [54]) of the scale (Table 3). Polychoric correlations among the items ranged from .284 to .546 in the ChYMH sample and from .208 to .448 in the ChYMH-DD sample with the exception of the item reflective of nightmares where the correlation was .140. Correlations were weaker for children with developmental/intellectual disabilities in comparison to those without such disabilities. Despite this, the correlations were all positive and none of the correlations have a 95% confidence interval that contains 0, indicating significant correlations among all the items in both samples. See Table 4 for the polychoric correlations among the Anxiety Scale items for the ChYMH and the ChYMH-DD samples.

**Table 4:** Polychoric correlations (95% CI) among the Anxiety Scale items

	Polychoric Correlation (*95% CI*)					
	1.	2.	3.	4.	5.	6.
ChYMH Sample						
1. Anxious complaints/ concerns	–					
2. Unrealistic fears	.546 (.519–.575)	–				
3. Obsessive thoughts	.461 (.413–.478)	.479 (.458–.515)	–			
4. Intrusive thoughts or flashbacks	.297 (.267–.340)	.388 (.351–.421)	.466 (.433–.501)	–		
5. Episodes of panic	.424 (.402–.459)	.496 (.461–519)	.454 (.420–.483)	.459 (.428–.491)	–	
6. Nightmares	.293 (.269–.334)	.398 (.364–.452)	.284 (.237–.307)	.435 (.404–.470)	.345 (.308–.380)	–
ChYMH-DD Sample						
1. Anxious complaints/ concerns	–					
2. Unrealistic fears	.448 (.352–.532)	–				
3. Obsessive thoughts	.351 (.256–.446)	.263 (.156–.363)	–			
4. Intrusive thoughts or flashbacks	.208 (.097–.301)	.356 (.250–.459)	.325 (.212–.420)	–		
5. Episodes of panic	.292 (.198–.384)	.453 (.362–.543)	.293 (.182–.381)	.432 (.315–.523)	–	
6. Nightmares	.148 (.043–.256)	.304 (.196–.399)	.140 (.037–.246)	.440 (.335–.552)	.255 (.156–.365)	–

An unrestricted factor analysis was then conducted to examine the number of latent variables that underlie the scale items. Polychoric correlations among the six items were used as opposed to Pearson’s correlations to account for the ordinal nature of the data [60]*.* The Kaiser-Meyer-Olkin (KMO) test was 0.80 in the ChYMH sample and 0.71 in the ChYMH-DD sample, suggesting that the scale items are suitable for unrestricted factor analysis. Using the unrestricted factor analysis based on the polychoric correlations among the scale items, only one factor was extracted from the data in both samples; all items loaded on the first unrotated factor, with the factor loadings ranging from 0.52 to 0.74 in the ChYMH sample and from 0.44 to 0.69 in the ChYMH-DD sample (Table 5). The first factor explained 51.45% of the total variance in the ChYMH sample and 43.18% of the total variance in the ChYMH-DD sample. The standardized Cronbach’s alpha for the Anxiety Scale, also calculated based on polychoric correlations, was 0.81 in the ChYMH sample and 0.73 in the ChYMH-DD sample. See Table 6 for the descriptive statistics for all the items of the Anxiety scale in both samples.

**Table 5:** Unrotated factor loadings and communalities for the Anxiety Scale items

Item	ChYMH Sample	ChYMH- DD Sample		
	Factor Loading	Communality	Factor Loading	Communality
1. Anxious complaints/ concerns	.643	.414	.525	.276
2. Unrealistic fears	.743	.551	.690	.477
3. Obsessive thoughts	.673	.453	.476	.227
4. Intrusive thoughts or flashbacks	.624	.389	.616	.379
5. Episodes of panic	.678	.459	.634	.401
6. Nightmares	.521	.271	.443	.196

**Table 6:** Descriptive statistics for the Anxiety Scale items

	Mean	95% CI	Variance	Skewness	Kurtosis	Item-Total Correlation
ChYMH Sample						
1. Anxious complaints/ concerns	1.452	1.40–1.50	2.415	0.536	−1.276	.739
2. Unrealistic fears	0.974	0.93–1.02	1.839	1.151	−0.071	.693
3. Obsessive thoughts	0.970	0.92–1.02	2.009	1.154	−0.197	.672
4. Intrusive thoughts or flashbacks	.462	0.43–0.49	0.879	2.253	4.512	.494
5. Episodes of panic	.744	0.71–0.78	1.140	1.499	1.542	.622
6. Nightmares	.582	0.55–0.61	0.880	1.841	3.094	.493
ChYMH-DD Sample						
1. Anxious complaints/ concerns	1.583	1.42–1.75	2.735	0.385	−1.524	.693
2. Unrealistic fears	0.837	0.71–0.97	1.679	1.398	0.631	.594
3. Obsessive thoughts	1.351	1.18–1.52	2.774	0.666	−1.296	.667
4. Intrusive thoughts or flashbacks	0.266	0.20–0.34	0.494	3.166	10.773	.415
5. Episodes of panic	0.742	0.63–0.86	1.294	1.534	1.382	.560
6. Nightmares	0.389	0.31–0.47	0.592	2.389	6.091	.303

### Convergent validity

#### CBCL internalizing clinical group membership

**Fig. 1:**
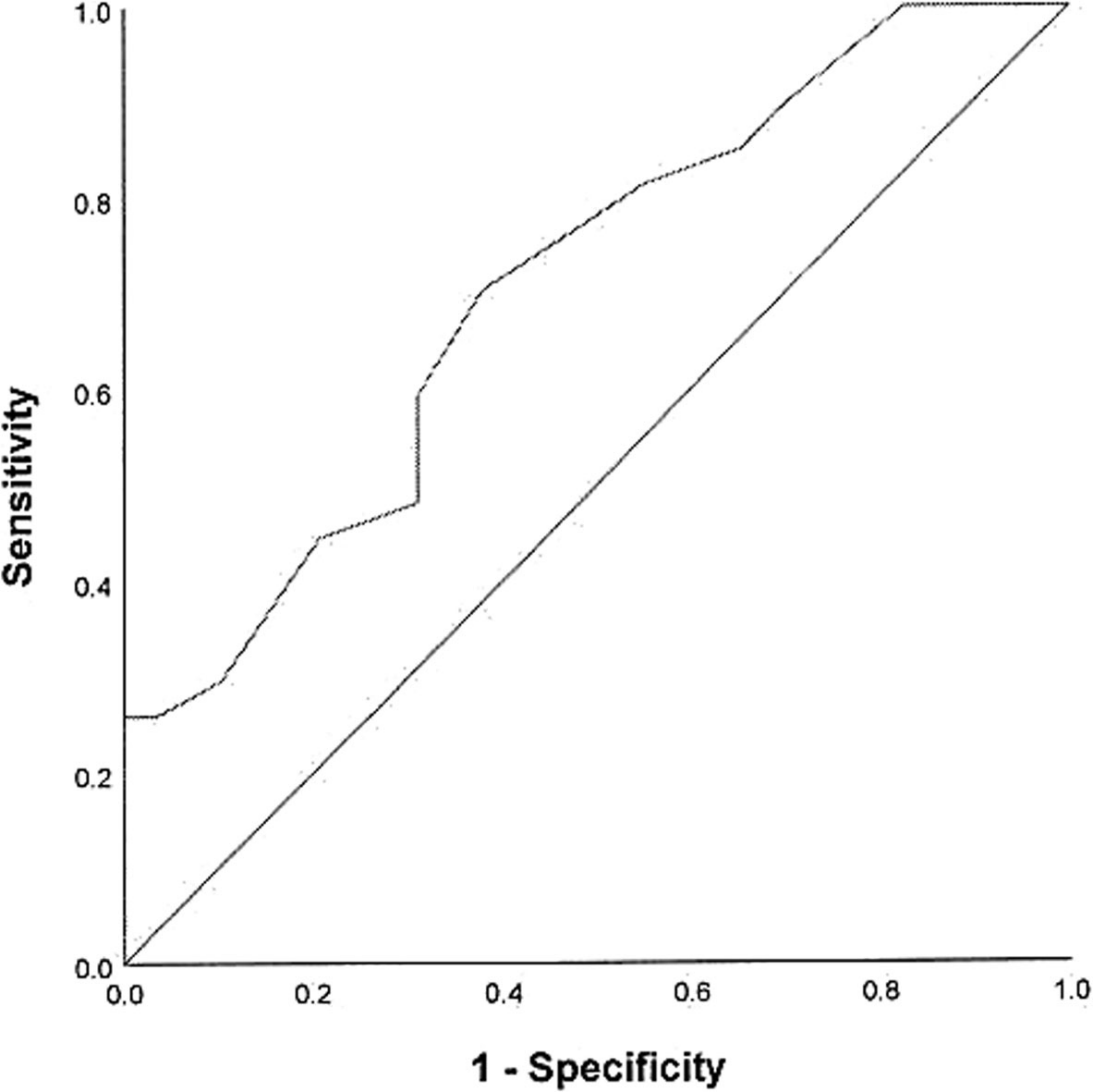
ROC curve for anxiety scale score for the prediction of CBCL internalizing score. The children’s scores on the Anxiety Scale were used to predict whether their CBCL internalizing t-score was within the clinical range or not. AUC = .709 (*SE =* .068), *p* = .007; Asymptotic 95% CI = .575–.843

Among the 57 children in the ChYMH sample who were assessed with the CBCL, 27 had an internalizing t-score of 70 or above (within the clinical range), 29 had a t-score below 70 (not in the clinical range), and the score was missing for the remaining one child. The children’s scores on the Anxiety Scale were used to predict whether their CBCL internalizing t-score was within the clinical range or not. The Area Under the Curve (AUC) for the ROC analysis was significant, AUC = .709 (*SE =* .068), *p* = .007, Asymptotic 95% CI = .575–.843 (Fig. 1). Pythagorean’s Index was used to determine appropriate cut-off points and provided a range for both sensitivity and specificity [61, 62]. Here, Pythagorean’s Index suggested a cut-off point of 5.0 for the Anxiety Scale, which corresponded to a sensitivity ranging from 70.4 to 81.5% and a specificity ranging from 44.8 to 62.1% in predicting a CBCL internalizing score that falls within the clinical range.^2^ The bivariate correlation between the children’s scores on the Anxiety Scale and their CBCL internalizing t-scores was *r* = .444, *p* < .001.

#### DSM-IV anxiety disorders diagnoses

**Fig. 2:**
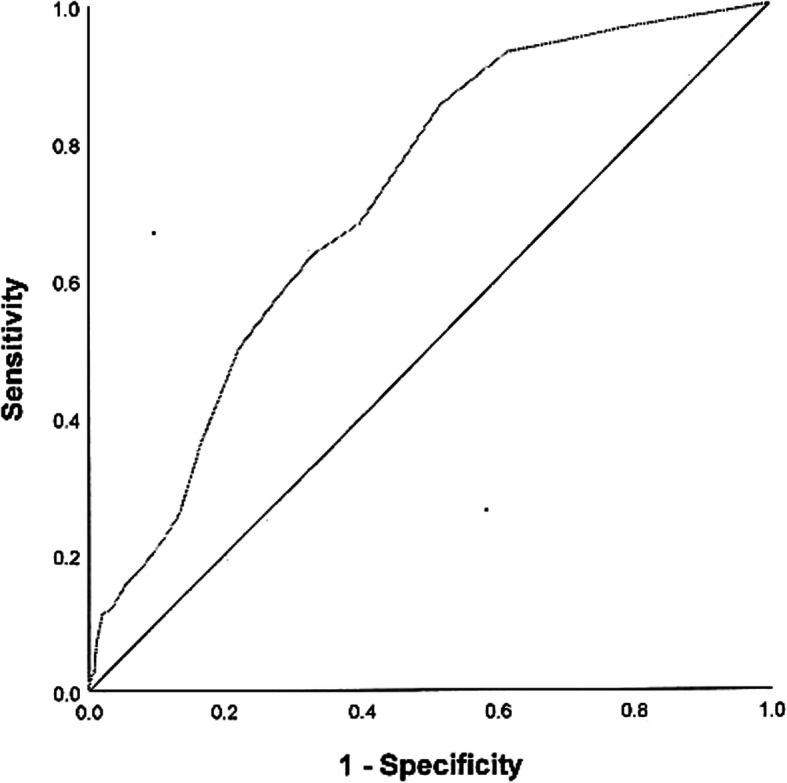
ROC curve for anxiety scale score for the prediction of DSM-IV Anxiety Disorder diagnosis. The children’s scores on the Anxiety Scale were used to predict clinical diagnoses of anxiety. AUC = .714 (*SE* = .025), *p* < .001; Asymptotic 95% CI = .665–.763

The convergent validity was examined against the DSM-IV Anxiety Disorders diagnoses in the ChYMH-DD sample. The ROC analysis generated an AUC of .714 (*SE* = .025), *p* < .001, Asymptotic 95% CI = .665–.763 (Fig. 2). An optimal cut-off score of 5.0 was determined according to Pythagorean’s Index, which.

corresponded to a sensitivity ranging from 63.8 to 68.1% and a specificity ranging from 60.3 to 68.9% in predicting the clinical diagnoses in anxiety disorders.

#### BASC-3 anxiety scores

A correlation was calculated between the total scores on the Anxiety Scale and the BASC-3 Anxiety *t*-scores using the ChYMH-Screener sample. Among the 72 children who had data available for both scales, the correlation was positive and significant with a large effect size, *r* = .517, *p* < .001.

## Discussion

Anxiety is one of the most prevalent psychiatric conditions in childhood [23] and the accuracy and efficiency of its assessment is of the upmost importance. Evidence suggests that early identification and assessment are critical to improving the lives of individuals with chronic anxiety and other internalizing symptomologies. The present study therefore aimed to establish the validity of the Anxiety Scale on the interRAI child and youth assessment instruments, including the ChYMH, ChYMH-DD, and ChYMH-Screener. The Anxiety Scale is being utilized in clinical settings and is embedded within the larger assessments to provide a comprehensive, holistic evaluation of children’s mental and physical health, as well as behavioural and emotional functioning. Establishing this scale’s psychometric properties provides further supporting evidence to a larger collection of literature regarding the ChYMH assessment qualities [32–36]. Moreover, given the challenges of assessing co-morbid psychiatric conditions in children with intellectual and developmental disabilities, inclusion of a unique sample with developmental disability was a crucial step towards effective assessment of anxiety in this vulnerable population.

To begin this examination, we assessed scale reliability in each sample using inter-item reliability. Polychoric correlations between the Anxiety Scale items were significant, both with the ChYMH and ChYMH-DD samples. A factor analysis further demonstrated that all of the items load onto a single factor in each sample, implying that not only are the items capturing the intended construct, but they are also functioning as a cohesive, integrated scale.

It should be noted that *nightmares* were more weakly associated with other items on the Anxiety Scale. While several studies have demonstrated the association between nightmares and anxiety [63, 64], other studies have failed to find such an association [65, 66]. This may account for the weaker correlation and may be further complicated by the fact that children with intellectual disabilities may also struggle with communicating and verbalizing their nightmares. However, given that nightmares have been associated with pathological symptoms of trait anxiety [67–69], as well as internalizing disorders in childhood [70], this item was included within the scale. Items (e.g., anxious complaints or concerns reflected through worry, unrealistic fears, obsessive and intrusive thoughts, as well as episodes of panic) were developed and included in this scale based on key symptoms identified in the literature related to anxiety disorders as well as based on expert clinical consultation. Despite the fact that the "nightmares" item correlates less strongly with other items on the scale, the Anxiety Scale demonstrates adequate internal consistency overall and is a reliable component of the ChYMH instruments.

Next, the convergent validity of the Anxiety Scale was tested using CBCL internalizing scores, DSM-IV Anxiety Disorders diagnosis, and BASC-3 Anxiety t-scores for each of the three samples, respectively. In the ChYMH subsample, a ROC analysis found that children’s scores on the Anxiety Scale significantly predicted whether their CBCL internalizing scores fell within the clinical range (t-scores> = 70) or not. The AUC value of .709 suggested that a child randomly selected from those who display clinical levels of internalizing behaviours, according to the CBCL, will score higher 70.9% of the time than a child randomly selected from those whose CBCL internalizing score does not fall within the clinical range. This finding indicates a moderate accuracy of the Anxiety Scale score in predicting the CBCL internalizing clinical group membership [71]. It should be noted that the specificity at predicting the clinical group membership was lower than sensitivity for the ChYMH sample. Specifically, sensitivity ranged from 70.4–81.5% suggesting that a cut-point of 5 would be sensitive enough to capture the majority of the cases. However, specificity, or the ability to identify children who truly have anxiety was lower than sensitivity. Given that sensitivity and specificity are inversely proportional to one another, more false positives than false negatives would result when utilizing this cut-point. An important consideration is that sensitivity and specificity may have been negatively impacted by the fact that the CBCL internalizing scale represented a *broad band* domain that incorporates withdrawn/depressed, anxious/depressed, and somatic complaints rather than a specific scale that only represents the construct of anxiety.

Meanwhile, in the ChYMH-DD sample, children’s scores on the Anxiety Scale also significantly predicted whether they were diagnosed with DSM-IV Anxiety Disorders or not. The AUC value of .714 indicated that, for this specific population, a child randomly chosen from those who have a DSM-IV Anxiety Disorders diagnosis will score higher on the Anxiety Scale 71.4% of the time than a child randomly chosen from those who do not have an Anxiety Disorders diagnosis. Similar to the other results, specificity was lower than sensitivity. Previous research has indicated that it can be much more difficult to diagnose anxiety in these children due to their limited language and communication abilities. A variety of diagnostic challenges have been noted in the literature [72] when diagnosing children with developmental disabilities, which is further complicated by the fact that they may not display age-typical symptoms of anxiety, and often struggle to articulate their internal state [73]. Consequently, a gold standard criterion may require a combination of tests (e.g., standardized parent, teacher, child report as well as observation in concert with a diagnosis of anxiety), administered all within a strict time period to provide a more accurate model for diagnostic decision making.

Further, the CBCL findings are well supported by the BASC-3 results, which also demonstrated a positive and significant correlation between the BASC-3 anxiety scale and the Anxiety Scale on the interRAI ChYMH. The ChYMH-Screener sample showed a large, positive correlation with a large effect size between their scores on the Anxiety Scale and the BASC-3 anxiety t-scores, providing further evidence in support of this scale’s convergent validity when tested against a well-established measure. This observed convergent validity for both the ChYMH and ChYMH-DD samples also speak to the appropriateness and clinical utility of this scale in applied settings, as it shows consistent screening ability in both sub-populations.

With respect to symptom capturing, the interRAI Anxiety scale has a few advantages over other measures of pediatric anxiety such as that captured by the CBCL and the BASC. For example, the CBCL is completed by parents, whereas the Anxiety Scale on the interRAI ChYMH is completed by the clinician, using multi-informant data. This disagreement between the various sources might also have contributed to the moderate, rather than high, correlation between children’s CBCL internalizing scores and Anxiety Scale scores. Previous research on assessment of child anxiety symptoms has found a discordance between parents’ and children’s own reports and stressed the need for a multi-informant approach in assessing anxiety among children [74–76]. Moreover, anxiety is difficult to assess because, in many cases, it is quite covert, and its underlying symptoms can be rather subjective (e.g., negative thoughts). The ChYMH uses all available information including reports of the child, parent, teacher, assessor, as well as collateral information; this allows integration of multiple observations of anxious behaviour. It also assesses the frequency, intensity, and severity of the symptoms. While many anxiety scales rely on the child’s self-reported symptoms, this can be impacted significantly by their cognitive and emotional development, which in turn, impacts the quality of responses [77]. Therefore, utilizing a clinician-based assessment (like the interRAI system) reduces the social desirability effect, where anxious children may feel embarrassed or uncomfortable reporting their feelings or thoughts [78]. It also combines the most comprehensive information from a variety of sources to get a well-rounded, accurate picture of anxious symptomology. In addition, measures such as the CBCL combine a variety of symptoms such as social withdrawal, preference for being alone, not speaking in social situations, and gaze avoidance; common features of Autism Spectrum Disorder (ASD). Children with developmental disabilities, such as ASD, might show elevated social anxiety, especially when facing increasingly complex social situations starting from adolescence [24]. Anxiety measures that include a subscale assessing social skills (e.g., eye gaze) might therefore overestimate the actual anxiety symptoms of children with developmental disabilities. In comparison, the Anxiety Scale is embedded into the interRAI ChYMH and ChYMH-DD assessment tools, which employ multi-modal methods of assessment to help differentiate core ASD symptoms from comorbid anxiety disorders, an approach that is viewed as more comprehensive given that clinician ratings have clinically important information that is not available through parent or self-report measures alone that is unique to prognosis [79].

Overall, the Anxiety Scale was examined in three different samples (developmental, mental health, and school/community sample) with results suggesting that it has generalizability across a variety of children with diverse care needs. Additionally, unlike other scales, a combination of self-report and interview data, observed behaviour/arousal, and multiple informant data have been reported to be the best approach to identify anxiety-based symptomatology in children with developmental disabilities, especially ASD [80]. The interRAI Child and Youth suite of instruments utilizes this comprehensive, multi-informant approach to evaluate anxiety and related symptoms in young, vulnerable populations. However, it is important to note that this scale is not intended to replace other comprehensive anxiety measures, and while it has clinical utility in the context of the ChYMH assessments, is not intended to be used as a *standalone* measure for anxiety disorder identification. This scale while reliable, has a limited number of items, and should be utilized as a part of the ChYMH’s comprehensive assessment of functioning.

### Limitations

There are a few limitations of this study that should be acknowledged. First, with respect to anxiety diagnoses, high co-morbidities contribute to the challenging nature of formulating diagnoses of children with internalizing disorders, as other co-morbid disorders might be more difficult to differentiate from one another [81]. This difficulty is further complicated by communication issues that are often present in children with intellectual disabilities (e.g., expressive and receptive language weaknesses).

With respect to the assessment process, it is possible that an assessor may be faced with disparate information between the various sources, and therefore, must determine the *best* response based on the conflicting reports. This can be particularly difficult as it involves determining how to weigh the various reports, understanding that behaviour may change across different contexts, and then integrate all the clinical information into one final score that is the most accurate and representative of the child’s functioning and behaviour.

While examining the utilization of the Anxiety Scale across three diverse samples (high-risk school sample, mental health, and intellectual disabilities) was a strength, all validation data (e.g., actual diagnosis, CBCL, BASC) was not available across all groups. This was a result of the fact that the services provided for children who participated in the study varied across service sectors. As a result, several children within the database may have been unable to obtain access to diagnosticians or other health care practitioner with the ability to communicate the controlled act of a diagnosis. Consequently, several children may have met the diagnostic criteria for an anxiety disorder but did not receive the diagnosis due to the limited access to specialized services (e.g., access to a psychiatrist, psychologist). This, in turn, would have significantly reduced our validation sample. Therefore, we were unable to compare and contrast the same validation measures across all samples. In addition, the sample sizes for the CBCL and BASC-3 samples were small, and therefore the conclusions drawn from this data must be considered carefully. And as previously mentioned, the CBCL is a global internalizing scale, and in the future, a validated scale specifically measuring anxiety only, (rather than utilizing a global internalizing scale), should be utilized to further validate the scale.

Finally, generalizability is limited to children and youth attending school in a high-risk area or for those children who have been referred for mental health services. At this point in time, generalization to the broader community beyond these samples are not possible.

### Summary

This study examines the psychometric properties of the Anxiety Scale among children. The Anxiety Scale is a 6-item scale assessing the symptoms of anxiety and is embedded in the ChYMH, ChYMH-DD, and ChYMH-Screener of the interRAI child and youth assessment tools. Despite the limitations noted above and the potential areas for future research, this study broadened the psychometric support for the interRAI ChYMH to a wider population of children and youth, including those with developmental and intellectual disabilities. The study results suggest that the Anxiety Scale is a valid scale within the instruments, exhibiting clinical utility. These findings provide additional support to a larger body of research surrounding the interRAI measures, which show consistent psychometric rigour [32–36].

Importantly, this study also includes a sample of children with developmental/intellectual disability. Intellectual disability is considered a lifetime condition and can be extremely challenging for the child and caregivers who support them [30]. Children with these delays often experience increased anxiety as they get older and begin to understand and perceive the disparities in social and cognitive abilities, compared to other age-mates [82, 83]. The ChYMH-DD is a comprehensive and, importantly, context-specific assessment designed explicitly for children with developmental/intellectual disabilities. Therefore, the observed validity of scales is essential to ensuring that children with these unique needs are appropriately assessed.

## Conclusions

Results suggest that the Anxiety Scale is a valid scale, which exhibits clinical utility in children with and without developmental disabilities. This finding contributes to the existing literature which supports the use of interRAI ChYMH assessments for the evaluation of children’s mental and physical health concerns. In addition, unlike other assessment systems, interRAI instruments can be utilized across service sectors as well as longitudinally as a child ages due to identical core items across the family of interRAI instruments. This data can also be compared to other diagnostic information or professional insight, used often to provide corroborating evidence in multiple service sectors across the lifespan. Consequently, many countries are now utilizing the interRAI assessment system as a health information system to develop case-mix systems for more appropriate allocation of resources (e.g., determining eligibility for support services and assisted-living programs) [36].

In summary, the development of the interRAI instruments allows opportunities for assessment and outcome measurement across developmental stages [16, 31, 32]. This research findings reported herein provides empirical support to the larger body of literature that illustrates the utility of the interRAI suite of instruments for a variety of health-related issues, including anxiety [35]. The use of these assessments over several developmental stages enables clinicians and mental health service providers to facilitate more efficient assessment, care planning, and prioritization, while also providing a framework for fostering optimal outcomes for vulnerable children, youth and families.

## Data Availability

Due to the highly sensitive and confidential nature of the data, as well as the ethical requirements required for use of such data at the present institution (i.e., data collected on secure server, VPN protected, password protected data in secure room with no access to internet or USB ports, etc.), data will not be made freely available. Moreover, participating mental health agencies required that data not be made freely accessible, to protect the anonymity of participants.

## Abbreviations

ASD: Autism Spectrum Disorder
AUC: Area Under the Curve BASC-3: Behaviour Assessment System for Children, 3rd Edition
IDD: Intellectual and developmental disabilities (IDD)
ChYMH: InterRAI Child and Youth Mental Health Instrument
ChYMH-DD: InterRAI Child and Youth Mental Health and Developmental Disability
ChYMH-Screener: InterRAI Child and Youth Mental Health-Screener assessment
CAPS: Collaborative action plans
CBCL: Child Behaviour Checklist
DSM-IV-TR: Diagnostic and Statistical Manual of Mental Disorders, 4th Edition, Text Revision
DWSL: Diagonally weighted least squares
KMO: Kaiser-Meyer-Olkin
ROC: Receiver operating characteristic

## Footnotes

1 Please note: Many children seeking services at mental health agencies do not receive a diagnosis (e.g, do not meet the criteria or do not have access to health care professionals able to diagnose); therefore, in those cases, no DSM data could be captured and subsequently, is reflected in Tables 1 and 2.

2 The sensitivity and specificity were calculated at 1-point increment starting from 0.5 in the ROC analysis (i.e., when the Anxiety score was 0.5, 1.5, 2.5, etc.). Pythagorean’s Index was calculated based on each set of sensitivity and specificity. As the actual score on the Anxiety Scale can only be integers, we chose the integer that was 0.5 point below the one associated with the highest Pythagorean’s Index score as our cut-off point. We therefore report the sensitivity and specificity as ranges, with the values associated with the closest points to the cut-off score as the upper and lower bounds of the ranges.

## References

[1] Mental Illness and Addictions: Facts and Statistics. Canadian Mental Health Association. 2017. www.camh.ca/News_events/Key_CAMH_facts_for.../addictionmentalhealthstatistics.html Accessed 24 Feb 2019.

[2] Luby JL, Si X, Belden AC, Tandon M, Spitznagel E. Preschool depression: Homotypic continuity and course over 24 months. Arch Gen Psychiat. 2009. 10.1001/archgenpsychiatry.2009.97. 10.1001/archgenpsychiatry.2009.97 PMC3184302 19652129

[3] McLeod GFH, Horwood LJ, Fergusson DM. Adolescent depression, adult mental health and psychosocial outcomes at 30 and 35 years. Psychol Med. 2016. 10.1017/S0033291715002950. 10.1017/S0033291715002950 26818194

[4] Veldman K, Reijneveld SA, Ortiz JA, Verhulst FC, Bültmann U. Mental health trajectories from childhood to young adulthood affect the educational and employment status of young adults: results from the TRAILS study. J Epidemiol. 2015. 10.1136/jech-2014-204421. 10.1136/jech-2014-204421 25667302

[5] Bufferd SJ, Dougherty LR, Carlson GA, Rose S, Klein DN. Psychiatric disorders in preschoolers: continuity from ages 3 to 6. Am J Psychiatry. 2012. 10.1176/appi.ajp.2012.12020268. 10.1176/appi.ajp.2012.12020268 PMC3513401 23128922

[6] Smith JP, Smith GC. Long-term economic costs of psychological problems during childhood. Soc Sci Med. 2010. 10.1016/j.socscimed.2010.02.046. 10.1016/j.socscimed.2010.02.046 PMC2887689 20427110

[7] Waddell C, McEwan K, Shepherd CA, Offord DR, Hua JM. A public health strategy to improve the mental health of Canadian children. Can J Psychiatr. 2005. 10.1177/070674370505000406. 10.1177/070674370505000406 15898462

[8] Children’s Mental Health Ontario (2016). Ontario’s children waiting up to 1.5 years for urgently needed mental healthcare.

[9] Office of the Auditor General of Ontario (2016). Annual report 2016, volume 1. Toronto: Queen’s Printer for Ontario, Toronto.

[10] Maulik PK, Mascarenhas MN, Mathers CD, Dua T, Saxena S. Prevalence of intellectual disability: a meta-analysis of population-based studies. Res Dev Dis. 2011. 10.1016/j.ridd.2010.12.018. 10.1016/j.ridd.2010.12.018 21236634

[11] Emerson E, Hatton C. Mental health of children and adolescents with intellectual disabilities in Britain. Brit J Psychiat. 2007. 10.1192/bjp.bp.107.038729. 10.1192/bjp.bp.107.038729 18055952

[12] De Ruiter KP, Dekker MC, Verhulst FC, Koot HM (2007). Developmental course of psychopathology in youths with and without intellectual disabilities. J Child Psych and Psychiatry.

[13] Einfeld SL, Ellis LA, Emerson E (2011). Comorbidity of intellectual disability and mental disorder in children and adolescents: a systematic review. J Intellect Dev Dis..

[14] Foley KR, Dyke P, Girdler S, Bourke J, Leonard H (2012). Young adults with intellectual disability transitioning from school to post-school: a literature review framed within the ICF. Disabil Rehabil.

[15] Stewart SL, Currie M, Pearce J (2013). The interRAI child/youth mental health – developmental disability (ChYMH-DD) instrument. Schulich Med Dentistry, Clin Bull Dev Disabil Div.

[16] Stewart SL, Hirdes JP (2015). Identifying mental health symptoms in children and youth in residential and in-patient care settings. Healthcare management forum.

[17] Hirdes J, Smith T, Rabinowitz T, Yamauchi K, Pérez E, Telegdi N (2002). The resident assessment instrument-mental health (RAI-MH): inter-rater reliability and convergent validity. J Behav Health Ser R.

[18] Sattler J (2002). Assessment of children: behavioral and clinical applications.

[19] Briggs-Gowan MJ, Carter AS, Bosson-Heenan J, Guyer AE, Horwitz SM (2006). Are infant-toddler social-emotional and behavioral problems transient?. J Am Acad Child Psy.

[20] Halle TG, Darling-Churchill KE (2016). Review of measures of social and emotional development. J Appl Dev Psychol.

[21] Kjeldsen A, Janson H, Stoolmiller M, Torgersen L, Mathiesen KS (2014). Externalising behaviour from infancy to mid-adolescence: latent profiles and early predictors. J Appl Dev Psychol.

[22] Rose M, Devine J (2014). Assessment of patient-reported symptoms of anxiety. Dialogues Clin Neurosci.

[23] Bagnell AL (2011). Anxiety and separation disorders. Pediatr Rev.

[24] Roebuck R, Paquet M, Coultes-Macleod J (2008). Improving health outcomes for children and youth with developmental disabilities: A literature review in the health status of children and youth with developmental disabilities within a population health framework.

[25] White SW, Oswald D, Ollendick T, Scahill L (2009). Anxiety in children and adolescents with autism spectrum disorders. Clin Psychol Rev.

[26] Stewart SL, Falah Hassani K, Poss J, Hirdes J (2017). The determinants of service complexity in children with intellectual disabilities. J Intellect Disabil Res.

[27] Lapshina N, Stewart SL. Examining service complexity in children with intellectual disability and mental health problems who receive inpatient or outpatient services. J Intellect Dev Dis. 2018. 10.3109/13668250.2018.1440878.

[28] Bernabei R, Landi F, Onder G, Liperoti R, Gambassi G (2008). Second and third generation assessment instruments: the birth of standardization in geriatric care. J Gerontol A Biol Sci Med Sci.

[29] Hirdes JP, Frijters DH, Teare GF (2003). The MDS-CHESS scale: a new measure to predict mortality in institutionalized older people. J Am Geriatr Soc.

[30] Martin L, Hirdes JP, Fries BE, Smith TF (2007). Development and psychometric properties of an assessment for persons with intellectual disability—the interRAI ID. J Policy Pract Intel.

[31] Morris JN, Carpenter I, Berg K, Jones RN (2000). Outcome measures for use with home care clients. Can J Aging.

[32] Lau C, Stewart SL, Saklofske DH, Tremblay PF, Hirdes J (2017). 2017. Psychometric evaluation of the interRAI child and youth mental health disruptive/aggression behaviour scale (DABS) and hyperactive/distraction scale (HDS). Child Psychiat Hum D.

[33] Phillips CD, Patnaik A, Dyer JA, Nasier E, Hawes C, Fournier CJ, et al. Reliability and the measurement of activity limitation (ADLs) for children with special health care needs (CSHCN) living in the community. Disabil Rehabil. 2011. 10.3109/09638288.2011.555596. 10.3109/09638288.2011.555596 21345002

[34] Phillips CD, Patnaik A, Moudouni DK, Nasier L, Dyer JA, Hawes C, et al. Summarizing activity limitations in children with chronic illness living in the community: a measurement study of scales using supplemented interRAI items. BMC Health Serv Res. 2012. 10.1186/1472-6963-12-19. 10.1186/1472-6963-12-19 PMC3280154 22270147

[35] Stewart SL, Hamza CA. The child and youth mental health assessment (ChYMH): an examination of the psychometric properties of an integrated assessment developed for clinically referred children and youth. BMC Health Serv Res. 2017. 10.1186/s12913-016-1970-9. 10.1186/s12913-016-1970-9 PMC5267403 28122563

[36] Stewart SL, Poss JW, Thornley E, Hirdes J (2019). Resource intensity for children and youth (RIChY): the development of an algorithm to identify high service users in children’s mental health. Health Serv Insights.

[37] Chan CL, Lai CK, Chi I (2014). Initial validation of the Chinese interRAI mental health in people with psychiatric illness. Int J Psychiat Clin.

[38] Kim H, Jung YI, Sung M, Lee JY, Yoon JY, Yoon JL (2015). Reliability of the interRAI long term care facilities (LTCF) and interRAI home care (HC). Geriatr Gerontol Int.

[39] Stewart S, Hirdes J, Curtin-Telegdi N, Perlman CM, McKnight M, MacLeod K (2015). interRAI Child and Youth Mental Health (ChYMH) Assessment Form and User’s Manual: For use with In-patient and Community-based Assessments.

[40] Stewart SL, LaRose L, Nicolson R, McKnight M, Knott W, Currie M (2015). interRAI Child and Youth Mental Health and Developmental Disability (ChYMH-DD) Assessment Form and User’s Manual: for Use with In-Patient and Community-Based Assessments. Version 9.3.

[41] Stewart SL, Hirdes JP, McKnight M (2015). interRAI Child and Youth Mental Health- Screener (ChYMH-Screener) Assessment Form and User’s Manual. Version 1.1.

[42] Stewart SL, Iantosca JM, Klassen J, Tucker M, Fisman S, McClean J (2017). interRAI 0–3 Assessment Form and User’s Manual. Version 1.

[43] Stewart SL, Theall LA, Morris JN, Berg K, Björkgren M, Declercq A (2015). interRAI Child and Youth Mental Health Collaborative Action Plans (CAPs): For Use with the Child and Youth Mental Health Assessment Instrument. Version 9.3.

[44] Arbeau K, Theall L, Willoughby K, Berman J, Stewart SL (2017). 2017. What happened? Exploring the relationship between trauma and provisional mental health diagnoses for children and youth. Psych..

[45] Achenbach TM, Edelbrock C. Child behaviour checklist. Burlington; 1991.

[46] Reynolds CR, Kamphaus RW. Behavior assessment system for children-third edition. Bloomington: Pearson; 2015.

[47] Gross D, Fogg L, Young M, Ridge A, Cowell JM, Richardson R, et al. The equivalence of the child behavior checklist across parent race/ethnicity, income level, and language. Psychol Assess. 2006. 10.1037/1040-3590.18.3.313. 10.1037/1040-3590.18.3.313 16953734

[48] American Psychiatric Association (2000). Diagnostic and Statistical Manual of Mental Disorders: DSM-IV-TR.

[49] Reynolds CR, Kamphaus RW (1998). Behavior assessment system for children manual.

[50] Weis R, Smenner L (2007). Construct validity of the Behavior Assessment System for Children (BASC) self-report of personality: evidence from adolescents referred to residential treatment. J Psychoeduc Assess.

[51] Doyle A, Ostrander R, Skare S, Crosby RD, August GJ (1997). Convergent and criterion-related validity of the behavior assessment system for children-parent rating scale. J Clin Child Psychol.

[52] Merrell KW, Blade RL, Lund J, Kempf KK (2003). Convergent and discriminant construct validity of the internalizing symptoms scale for children with the BASC-SRP-C. J Sch Psychol.

[53] Holgado-Tello FP, Chacón-Moscoso S, Barbero-García I, Vila-Abad E. Polychoric versus Pearson correlations in exploratory and confirmatory factor analysis of ordinal variables. Qual Quant. 2010. 10.1007/s11135-008-9190-y.

[54] Babakus E, Ferguson CE, Jöreskog KG (1987). The sensitivity of confirmatory maximum likelihood factor analysis to violations of measurement scale and distributional assumptions. J Marketing Res.

[55] Ferrando PJ, Lorenzo-Seva U, Chico E (2003). Unrestricted factor analytic procedures for assessing acquiescent responding in balanced, theoretically unidimensional personality scales. Multivariate Behav Res.

[56] Lorenzo-Seva U, Ferrando PJ (2020). Unrestricted factor analysis of multidimensional test items based on an objectively refined target matrix. Behav Res Methods.

[57] Mîndrila D (2010). Maximum likelihood (ML) and diagonally weighted least squares (DWLS) estimation procedures: a comparison of estimation bias with ordinal and multivariate non-normal data. Int J Digit Society.

[58] Lorenzo-Seva U, Ferrando PJ (2006). FACTOR: a computer program to fit the exploratory factor analysis model. Beh Res Methods.

[59] Zweig MH, Campbell G (1993). Receiver-operating characteristic (ROC) plots: a fundamental evaluation tool in clinical medicine. Clin Chem.

[60] Gadermann AM, Guhn M, Zumbo BD (2012). Estimating ordinal reliability for Likert-type and ordinal item response data: a conceptual, empirical, and practical guide. Pract Assess Res Eval.

[61] Freeman EA, Moisen GG. A comparison of the performance of threshold criteria for binary classification in terms of predicted prevalence and kappa. Ecol Model. 2008. 10.1016/j.ecolmodel.2008.05.015.

[62] Froud R, Abel G. Using ROC curves to choose minimally important change thresholds when sensitivity and specificity are valued equally: The forgotten lesson of Pythagoras. Theoretical considerations and an example application of change in health status. PLoS One. 2014;9:e114468. 10.1371/journal.pone.0114468 PMC4256421 25474472

[63] Abdel-Khalek AM. Reported nightmares and trait anxiety among Arab children, adolescents, and adults. J of Sleep Dis and Therapy. 2016. 10.4172/2167-0277.1000248.

[64] Zadra A, Donderi DC (2000). Nightmares and bad dreams: their prevalence and relationship to well-being. J Abnorm Psychol.

[65] Lancee J, Spoormaker VI, van den Bout J (2010). Nightmare frequency is associated with subjective sleep quality but not with psychopathology. Sleep Biol Rhythms.

[66] Wood JM, Bootzin RR (1990). The prevalence of nightmares and their independence from anxiety. J Abnorm Psychol.

[67] Minen JA, Barret M (2000). Nightmares and anxiety in elementary-aged children: is there a relationship?. Child Care Health Dev.

[68] Nielsen L, Laberge JP, Tremblay RE, Vitaro F, Montplaisir J (2000). Development of disturbing dreams during adolescence and their relation to anxiety symptoms. Sleep.

[69] Simor P, Kovacs I, Vargha A, Csoka S, Mangel B, Bodizs R (2008). Nightmares, dream anxiety and psychopathology: the validation of the Hungarian version of the Van anxiety scale. Psychiatria Hungarica: A Magyar Pszichiatriai Tarsasag Tudomanyos Folyoirata.

[70] Achenbach TM (1991). Manual for the child behavior checklist 4–18 and 1991 profile.

[71] Fischer JE, Bachmann LM, Jaeschke R (2003). A readers' guide to the interpretation of diagnostic test properties: clinical example of sepsis. Intens Care Med.

[72] Gjevik E, Eldevik S, Fjæran-Granum T, Sponheim E (2011). Kiddie-SADS reveals high rates of DSM-IV disorders in children and adolescents with autism spectrum disorders. J Autism Dev Disord.

[73] White S, Oswald D, Ollendick T, Scahill L (2009). Anxiety in children and adolescents with autism spectrum disorder. Clin Psychol Rev.

[74] Comer JS, Kendall PC (2004). A symptom-level examination of parent-child agreement in the diagnosis of anxious youths. J Am Acad Child Adolesc Psychiatry.

[75] Langer DA, Wood JJ, Bergman RL, Piacentini JC (2010). A multitrait–multimethod analysis of the construct validity of child anxiety disorders in a clinical sample. Child Psy & Hum Devel.

[76] Lipton MF, Augenstein TM, Weeks JW, De Los Reyes A (2014). A multi-informant approach to assessing fear of positive evaluation in socially anxious adolescents. J of Child Fam Studies.

[77] Grills AE, Ollendick TH (2003). Multiple informant agreement and the anxiety disorders interview schedule for parents and children. J Am Acad Child Ps.

[78] Silverman WK, Ollendick TH (2005). Evidence-based assessment of anxiety and its disorders in children and adolescents. J Clin Child Adolesc.

[79] Uher R, Perlis RH, Placentino A, Zvezdana Demovsek M, Henigsberg N, Mors O (2012). Self-report and clinician-rated measures of depression severity: can one replace the other?. Depress Anxiety.

[80] van Steelsen FJA, Bögels SM, Perrin S (2011). Anxiety disorders in children and adolescents with autistic spectrum disorders: a meta-analysis. Clin Child Fam Psych.

[81] Stark KD, Kaslow NJ, Laurent J (1993). The assessment of depression in children. J Emot Behav Disord.

[82] Bybee J, Zigler E, Burak JA, Hodapp RM, Zigler E (1998). Outerdirectedness in individuals with and without mental retardation: a review. Handbook of mental retardation and development.

[83] Dagnan D, Jahoda A (2006). Cognitive-behavioural intervention for people with intellectual disability and anxiety disorders. J Appl Res Intellect Disabil.

